# Supplementary material for: Autoantibody discovery across monogenic, acquired, and COVID19-associated autoimmunity with scalable PhIP-Seq
Source: bioRxiv. 2022 Mar 24:2022.03.23.485509. Preprint. [Version 1] doi: 10.1101/2022.03.23.485509 (PMC8963698; doi:10.1101/2022.03.23.485509)
Supplement: 1 [file NIHPP2022.03.23.485509V1-supplement-1.pdf]

bioRxiv preprint doi: https://doi.org/10.1101/2022.03.23.485509; this version posted March 25, 2022. The copyright holder for this preprint (which was not certified by peer review) is the author/funder, who has granted bioRxiv a license to display the preprint in perpetuity. It is made available under aCC-BY 4.0 International license.

Suppl. 1A

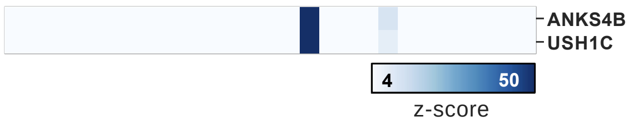

Suppl. 1B

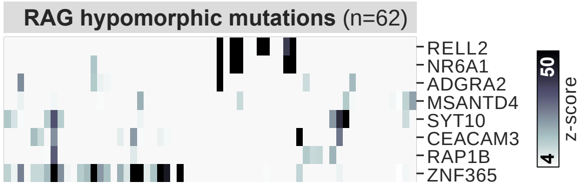

Figure 5 – Figure Supplement 1. A) PhIP-Seq has low detection sensitivity for known antigens USH1C and ANKS4B, but those patients with positive signal exhibit the previously reported coupled signal for both antigens. **B)** Additional, shared putative antigens within the cohort of RAG1/2-deficient patients (n=62).

bioRxiv preprint doi: https://doi.org/10.1101/2022.03.23.485509; this version posted March 25, 2022. The copyright holder for this preprint (which was not certified by peer review) is the author/funder, who has granted bioRxiv a license to display the preprint in perpetuity. It is made available under aCC-BY 4.0 International license.

SUPPLEMENTAL TABLES

Supplemental Table 1. Clinical metadata
